# An experimental study on the effects of the cortical thickness and bone density on initial mechanical anchorage of different Straumann® implant designs

**DOI:** 10.1186/s40729-021-00367-2

**Published:** 2021-08-30

**Authors:** Marie Emmert, Aydin Gülses, Eleonore Behrens, Fatih Karayürek, Yahya Acil, Jörg Wiltfang, Johannes Heinrich Spille

**Affiliations:** 1 Christian Albrechts University, Department of Oral and Maxillofacial Surgery, University Hospital of Schleswig-Holstein, Campus Kiel, Arnold-Heller-Straße 3, 24105 Kiel, Germany; 2 Department of Periodontology, Cankiri Karatekin University, Cankiri, Turkey

**Keywords:** Anchorage, Bone, Cortical, Implant, Primary, Stability

## Abstract

**Background:**

The aim of the current study was to comparatively assess the primary stability of different Straumann® implant designs (BLX, Straumann Tapered Effect, Bone Level Tapered, and Standard Plus) via resonance frequency analysis by using an implant insertion model in freshly slaughtered bovine ribs with and without cortical bone. Tapered Effect (4.1 × 10 mm), Bone Level Tapered (4.1 × 10 mm), Standard Plus (4.1 × 10 mm), and BLX (4.0 × 10 mm) implants were inserted into the distal epiphysis on the longitudinal axis of the freshly slaughtered bovine ribs. As a control, implants with the same sizes were inserted into the proximal diaphysis. The stability of the implants was examined with resonance frequency analysis.

**Results:**

BLX and Tapered Effect implants showed higher implant stability quotient values in both study and control groups. All implant systems showed a significant decrease of mechanical anchorage in the study group. BLX and Bone Level Tapered designs had a significantly lower loss of mechanical anchorage in the lack of cortical bone.

**Conclusion:**

Both Tapered Effect and BLX designs could ensure sufficient initial stability; however, BLX implants could be an appropriate option in the lack of cortical bone and poor bone quality at the implant recipient site.

**Clinical relevance:**

BLX is a novel implant system, which could be especially beneficial in the presence of spongious bone type at posterior maxillae.

## Introduction

The primary stability of a dental implant plays a key role in implant integration and long-term successful clinical outcome. Considering the implant recipient site, primary implant stability could be affected by several factors such as bone density, thickness of the cortical bone, and height of the alveolar ridge. In order to overcome these quantitative and qualitative deficits, various implant geometries and thread designs have been developed. Several studies comparing the outer geometry of the implants found that implants with conical shape [1] and/or tapered designs [2] had superior primary stability compared to those with a parallel shape and could significantly improve the initial mechanical anchorage.

It is well known that the loose structure of the trabecular bone could negatively affect mechanical anchorage [3–9]. Therefore, numerous clinical and experimental studies have mainly focused on the effects of bone density and geometry on implant stability. Nevertheless, the lack of cortical bone at the implant recipient site could be also challenging; thus, in addition to total bone thickness, the height of cortical bone along the implant has shown to have a relevant effect on primary implant stability [10, 11]. Sugiura et al. have suggested that the crestal cortical bone height could significantly affect the maximum extent of micromotion and peri-implant bone strain in the low-density cancellous bone under immediate loading [6]. Several studies have also clearly showed that improving primary stability via bi-cortical anchorage plays a great role in the success of immediate loading protocols such as All-on-4™ [12] and All-on-3 [13] and implant placements following sinus floor augmentation. However, 70% of all age-related bone loss is cortical [14] and the lack of cortical bone especially in the posterior maxillae could pose a great challenge for the clinician [15], especially in elderly dental implant patients.

The number of studies assessing the influence of cortical bone height on the mechanical anchorage regarding the dental implant design is limited [10, 11]. The aim of the current study was to comparatively assess the primary stability of different Straumann® implant designs (BLX, Tapered Effect [TE], Bone Level Tapered [BLT], and Standard Plus [SP]; Institute Straumann AG, Basel, Switzerland) via resonance frequency analysis by using an implant insertion model in freshly slaughtered bovine ribs with and without cortical bone.

## Material and methods

All experiments were performed at room temperature by two researchers (M.E. and A.G.). A total of 10 freshly slaughtered bovine ribs were used. Soft tissues and periosteum were completely removed off the bone.

**Fig. 1:**
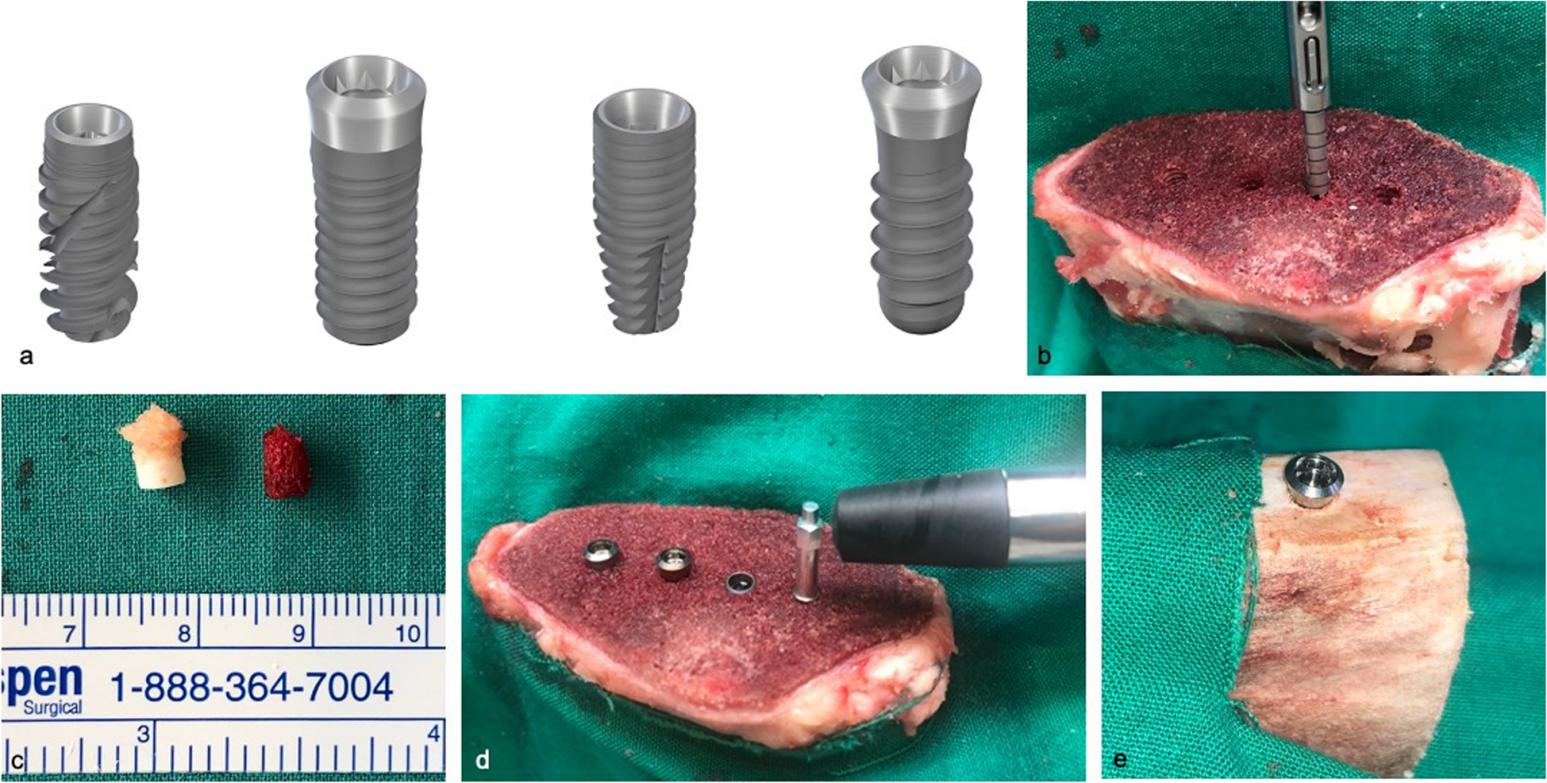
**a** From left to right: BLX (4.0 × 10 mm), Straumann Tapered Effect (4.1 × 10 mm), Bone Level Tapered (4.1 × 10 mm), and Standard Plus (4.1 × 10 mm). (The figures were taken from the product catalogue with kind permission of Institute Straumann AG, Basel, Switzerland © AG, 2015. All rights reserved.) **b** The implants were inserted into the distal epiphysis on the longitudinal axis of the freshly slaughtered bovine ribs. The distance between the implant shoulders was set to a minimum of 5 mm. **c** Prior to implant placement, bone biopsies were taken to histologically examine the cortical bone height at the implant recipient area. Left: Biopsy taken from the control, right: Study group. **d** Measurement of the primary stability of the implants with Osstell device inserted in cancellous bone (from left to right, Straumann Tapered Effect [4,1 x 10 mm], Standard Plus [4,1 x10 mm], Bone Level Tapered [4,1 x 10 mm], and BLX [4,0 x 10 mm]). **e**. Implants were also individually inserted into the proximal diaphysis of the bovine ribs to serve as control. (Tapered Effect 4.1 × 10 mm)

**Fig. 2:**
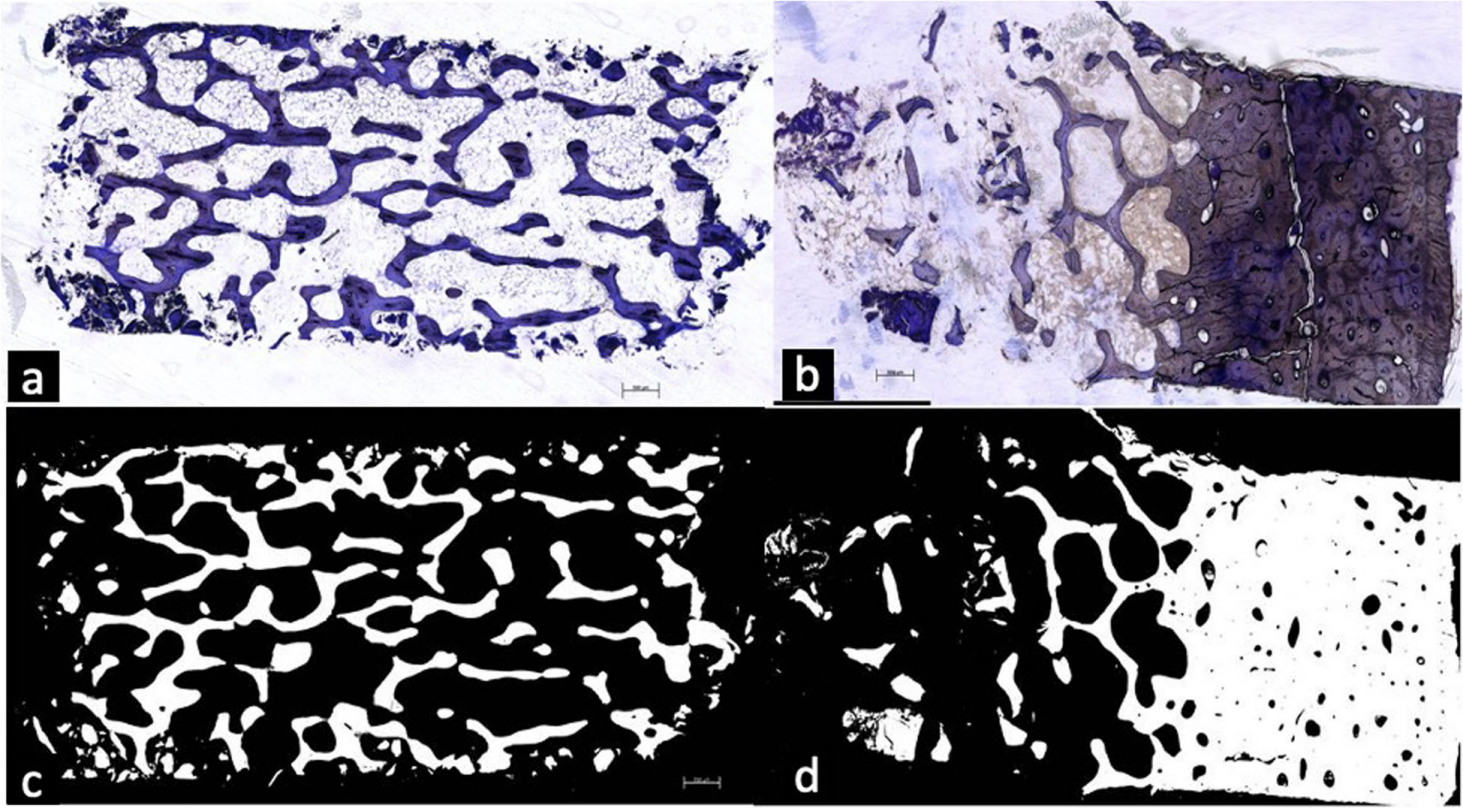
The height of the bone cortex was measured on samples stained with toluidine blue (**a**, **b**) and later by micro-radiography (**c**, **d**). **a** The study group. **b** The control group with the bone cortex. **c** The study group. **d** The control group with cortical bone

Straumann Tapered Effect (4.1 × 10 mm), Bone Level Tapered (4.1 × 10 mm), Standard Plus (4.1 × 10 mm), and BLX (4.0 × 10 mm) implants (Fig. 1) were inserted into the distal epiphysis on the longitudinal axis of the freshly slaughtered bovine ribs (Fig. 2). The distance between the implant shoulders was set to a minimum of 5 mm.

**Fig. 3:**
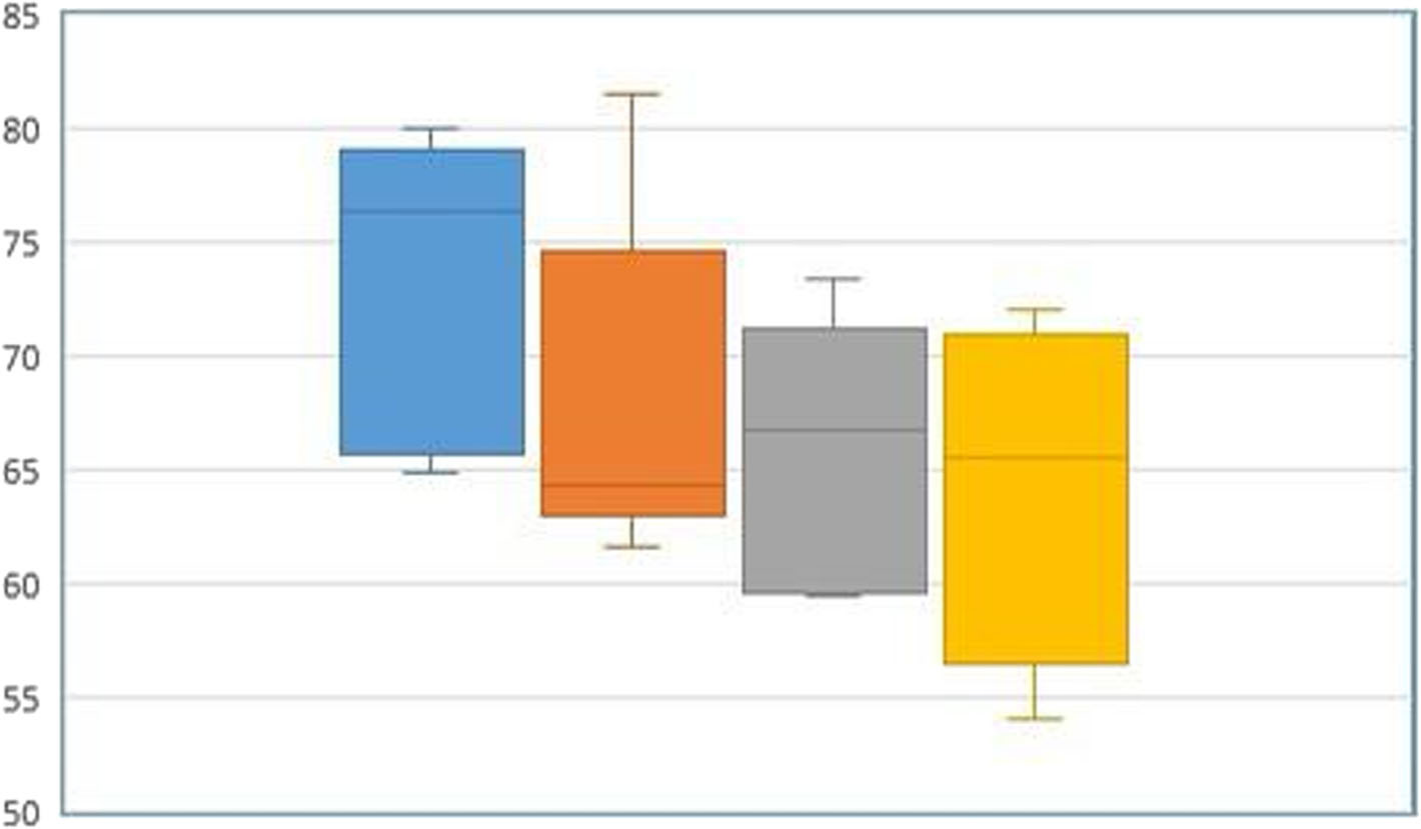
The comparative assessment of the ISQ values in type IV bone (SP [n 9], BLT [n 10], TE [n 7], and BLX [n 8]) in both groups (regardless of the presence of cortical bone) revealed that the BLX design had significantly higher mechanical anchorage compared to other implant systems (p 0.043)

**Fig. 4:**
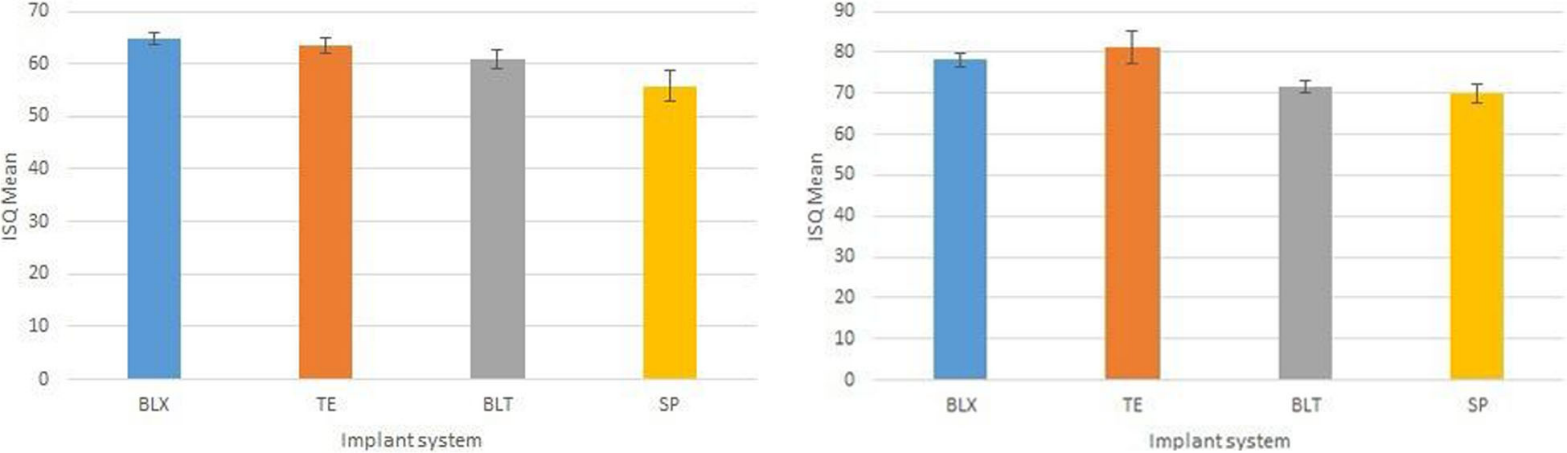
**a** BLX implant showed higher ISQ values compared to other groups. The significant difference was found when compared to SP and BLT groups (p 0.001 and p 0.0016, respectively). Besides that, TE implants showed also statistically significant superiority compared to SP (p 0.001). The difference between BLT and SP groups was also remarkable (p 0.003). **b** TE implants showed slightly higher values compared to BLX; however, the difference was statistically insignificant. BLX and TE implants showed significantly higher ISQ values compared to BLT and SP groups (BLX-BLT p 0.004, BLX-SP p 0.001 and TE-BLT p 0.001, TE-SP p 0.001, respectively)

As a control, implants were also individually placed into the proximal diaphysis of the bovine ribs, where a cortical bone surrounding the bone recipient site exists (Fig. 3). Implant insertions in both groups were conducted according to the drilling protocol with instrumentarium required for each system, with one exception: The use of both pilot and 2.0-mm drills were skipped; thus, prior to preparation of the samples, bone biopsies were taken with a trephine burr (2.3 mm) for histological evaluation to determine the cortical bone height at the corresponding implant recipient site (Fig. 4). For both groups, a total of 40 implant insertions were performed. Two blinded independent observers (E.B. and J. W.) have assessed the accuracy of placement. A peak insertion torque value of 30 N/cm has been exceeded by all implant insertions.

### Measurement of the primary stability

Implant stability quotient (ISQ) has been quantified by using resonance frequency analysis (RFA) via Osstell device (Osstell Mentor, Integration Diagnostics Ltd., Göteborg, Sweden). For each implant design, a suitable transducer was inserted into the implant body (SmartPeg, Osstell, Göteborg, Sweden). Measurements were taken in four different directions perpendicular to the SmartPeg, according to the manufacturer’s guidelines. The mean value of four measurements has been calculated to determine the final ISQ value of each implant. All measurements were conducted in triplicate.

### Histological evaluation

The tissue samples were histologically evaluated according to the technique described by Donath et al. [16] and later developed by Acil et al. [17, 18]. Briefly, tissue samples were placed into 10% neutral buffered formalin (NBF) for fixation for 4 days and embedded in methacrylate prior to sawing and grinding. Sawing and grinding were performed, and the samples were placed in glass vessels filled with monometric resin solution and incubated at 37–40 °C for 2 to 4 days for resin impregnation.

**Fig. 5:**
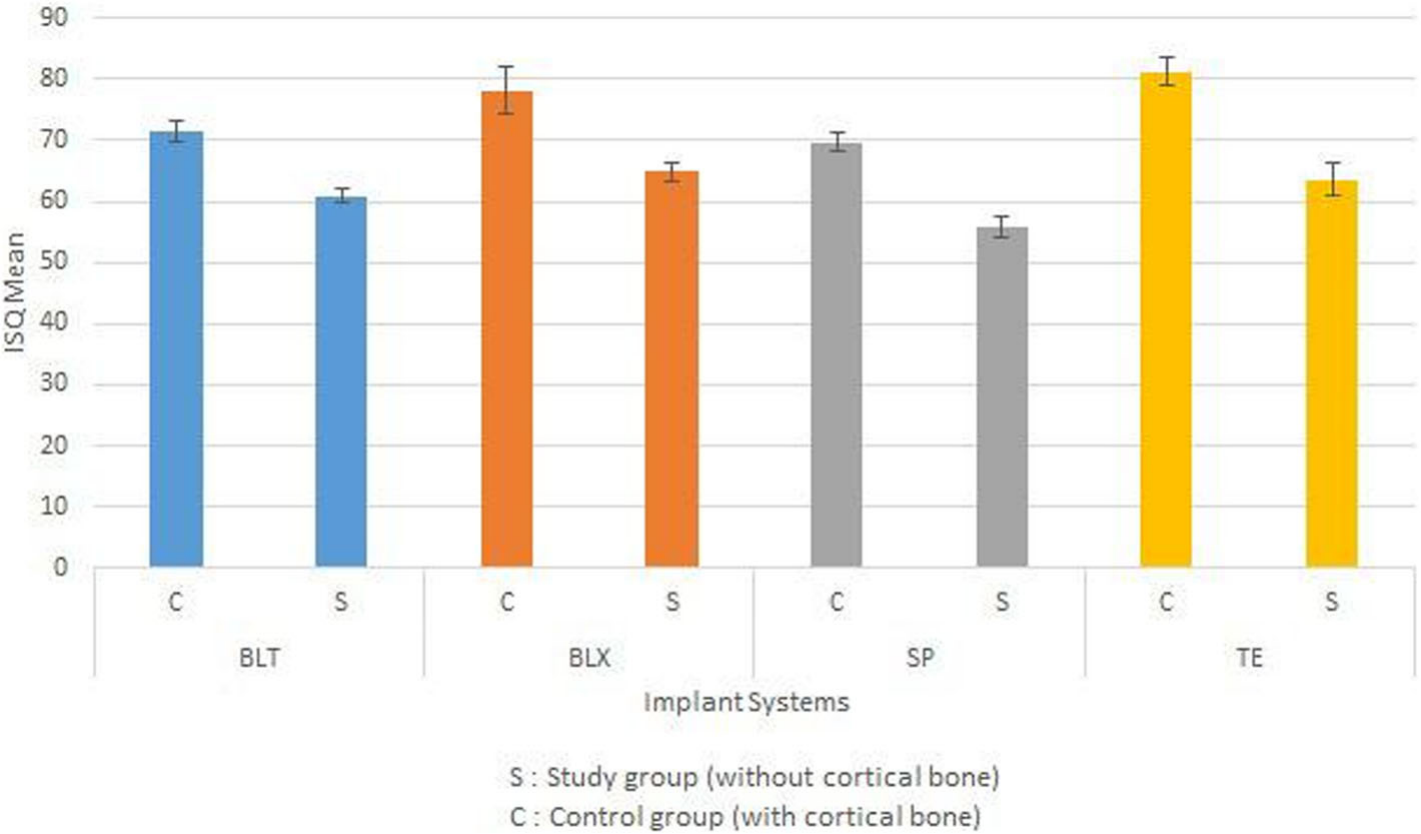
The decrease in percentage was lowest in the BLX group, followed by BLT. The difference was significant compared to the loss of mechanical anchorage obtained in the TE group (p 0.033 and p 0.008, respectively)

The samples were longitudinally cut with a band saw (Exakt, Norderstedt-Germany), and sections of about 100 μm were obtained via an oscillating diamond saw (Exakt, Norderstedt-Germany), grounded with the Sapphire 360 E grinder (ATM, Altenkirchen-Germany), and highly polished with silicon carbide paper (grades 500, 1200, 2400, and 4000). Staining was performed by using toluidine blue. The ground surface was decalcified with 0.1% formic acid and 20% methanol was applied for better cell and soft tissue staining. The samples were rinsed in distilled water and stained in a toluidine blue solution for 2 min. The height of the cortical bone was measured after micro-radiography of the specimens (Fig. 5). The preparations were digitally photographed with the Nikon photomicrography with a magnification of 509 and assessed manually with the Leica Q-Win Imaging program (McBain Systems, CA, USA). Relative amounts of bone area vs total tissue of the spongious bone were measured as described by Takahashi et al. [19] (% bone area/total tissue (% BA/TA)).

### Micro-radiography

The samples were taken from the slide and put on high-resolution micro-radiography plates in which the resolution is 2000 lines per millimeter (high-resolution plates, Kodak®, Rochester, NY 14650, USA). Subsequently, the exposure was performed in Faxitron 53855A (Hewlett-Packard, McMinnville, OR 97128, USA) at a focus distance of 16 cm. The voltage was set to 25 kV at a power of 3 mAs, exposure time of 6 min and 30 s for 70-μ samples, and up to 10 min and 30 s for 110-μm film thickness. The exposure time was increased by increments of 1 min per 10-μm thickness of the specimen. The plates were embedded for 5 min in Kodak® HRP developer at 20 °C under constant movement swung (HRP developers/distilled water, 1/3). In a 1% acetic acid bath, the development was stopped after 1 min. For fixation, Kodak® fixer 3000A was used for 10 min with agitation (Kodak® fixer 3000A/distilled water, 1/3). This was followed for 15 min by washing with water and a final rinse with Agepon® (400 mL of distilled water and 2 mL Agepon®) for 1 min. The plates were air dried and then covered with cover glasses (4 × 4.5 cm) using 1–2 drops of n-butyl acetate (xylene substitute) and a drop of Eukitt-air for 24 h. After a drying period of 7 days under an air extractor, the preparations were digitally photographed under a light microscope at a magnification of 1:18. The evaluation was performed using an image editing program (Adobe Photoshop 7.0 for Windows) (Fig. 2).

### Statistical analysis

Statistical software SPSS (IBM) was used for statistical analysis. Power analysis was performed to determine the smallest sample size that is suitable to detect the effect of a given test at the desired level of significance. The distribution of the variables was assessed by using the Shapiro-Wilk test. The comparison of two variables was performed via independent-T and paired-T tests. One-way analysis of variance (ANOVA) was conducted for comparative analysis of the variables. p < 0.05 was considered as statistically significant.

## Results

### Cortical bone height and trabecular structure

The average cortical bone height in the control group ranged between 2.34 and 3.21 mm (2.58 ± 0.615 mm). The histological examination revealed that, totally, n 6 (15.0%) of the bone structures correlated with type III bone (SP [n 1], TE [n 3], BLX [n 2]), whereas 34 (85.0%) of the specimens were identical to type IV bone (SP [n 9], BLT [n 10], TE [n 7], BLX [n 8]) [20]. The evaluation of the mean primary stability values of the implants inserted only in type IV bone in both groups (regardless of the presence of cortical bone) revealed that the BLX design had significantly higher mechanical anchorage compared to other implant systems (p 0.043) (Fig. 3).

### Comparison of implant designs

In the study group, the BLX implant showed higher ISQ values compared to other groups. The significant difference was found when compared to SP and BLT groups (p 0.001 and p 0.0016, respectively). Besides that, TE implants showed statistically significant superiority compared to SP (p 0.001). The difference between BLT and SP groups was also remarkable (p 0.003) (Fig. 4a).

In the control group, TE implants showed slightly higher values compared to BLX; however, the difference was statistically insignificant. BLX and TE implants showed significantly higher ISQ values compared to BLT and SP groups (BLX-BLT p 0.004, BLX-SP p 0.001 and TE-BLT p 0.001, TE-SP p 0.001, respectively) (Fig. 4b).

### Differences in ISQ values for each implant design regarding the presence of cortical bone

All implant systems showed a significant decrease of mechanical anchorage in the study group (BLX p 0.000*, TE p 0.001*, BLT p 0.000*, and SP p 0.002*, respectively). The decrease in ISQ values was assessed in percentages (Fig. 5). According to this, the decrease was lowest in the BLX group, followed by BLT. The difference was significant compared to the loss of mechanical anchorage obtained in the TE group (p 0.033 and p 0.008, respectively).

The results of the study revealed that the TE and BLX implants showed higher ISQ values compared to BLT and SP implants in both experimental and control groups.

## Discussion

In the literature, the influence of the height of the residual bone height on dental implant procedures has been widely studied. Despite several articles have showed that the height of the cortical bone at the implant recipient site could play a crucial role in the primary stability, the number of articles focusing on the effects of the cortical bone on the initial implant anchorage is limited [10, 11]. Miyamoto et al. have suggested that the initial stability at the time of implant installation is influenced more by cortical bone thickness than by implant length and is extremely important for implant stability at the time of surgery [4]. Similarly, Ming-Gene et al. [9] have stated that the bone strain is influenced more by the cortical bone thickness than by the implant length for immediately loaded implants.

According to an anatomical study performed by Katranji et al., the average cortical height of the edentulous posterior maxilla is about 2.06 mm [21]. It is also well known that the thickness of the compacta of the jawbones increases up to the age of 50 years and decreases significantly thereafter [22]. Additionally, the loss of the cortical bone presents a great challenge in the management of female dental implant candidates. Ko et al. [23] evaluated the prospective implant recipient sites by menopausal and post-menopausal women and showed that thickness of the crestal cortical bone was the lowest in the posterior maxilla with an average height of 0.66 ± 0.24 mm. Considering the experimental design (cortical bone of 0.00 mm vs 2.58 ± 0.615 mm) and type IV bone morphological structure, it might be proclaimed that the current model could sufficiently resemble the human posterior maxilla.

Romanos et al. [2] have previously compared the primary stability of different Straumann implants (BLT, SP, and TE) and found significantly higher implant stability for TE implants. The results of the current study were also in accordance with the existing literature. Besides that, all implant systems showed a significant decrease in ISQ values in the study group. However, despite the nearest ISQ values of BLX and TE implants in both study and control groups, it was remarkable that BLX implants showed significantly lower loss of the mechanical anchorage in the lack of cortical bone. In addition, BLT systems revealed lower ISQ values in both control and study groups; however, the decrease of the primary stability was also lower compared to TE and SP systems. This could be attributed to the conical outer design of the BLT implants and corresponds to the results of the previous articles [24, 25].

The BLX system is a novel “self-drilling” implant system developed by Straumann® (Straumann®, Institute Straumann AG, Basel, Switzerland). It has been proclaimed that its double thread design could provide an improved initial stability in all types of bone through uniform and controlled compaction and densification of the peri-implant bone [26]. The number of studies focusing on the primary stability of this novel system is limited [27].

I-Chiang et al. [7] have evaluated the micromotions of different implant types and found that the maximum stress of the peri-implant bone decreased as cortical bone thickness increased. According to that, the horizontal loading component induces stress concentration in the bone around the implant neck more easily than does the vertical loading component, especially by implants with a conical shape. In the literature, it has been clearly shown that TE implants presented higher stability values compared to the conventional implants, attributed to their compression to the cortical bone, which was also previously shown at implant recipient sites with poor bone quality by O’Sullivan et al. [28]. The superiority of the BLX system observed herein might be a result of its double thread design and the presence of two sharp and highly engaging threads at its apex, which allowed an advanced bone-to-implant contact and higher mechanical anchorage with minimal negative effect in the lack of the cortical bone. On the other hand, the loss of primary stability in the TE group could be due to the decreased compression of the implant neck to the surrounding bone, where the cortical bone does not exist.

It is well known that implant length and diameter could also influence the primary stability. Therefore, in the current study, implants with the same length and almost the same diameters were used. One of the main limitations of the current study is that the implant recipient site could exhibit different quantitative characteristics in its both cancellous and cortical structures [29]. To overcome this issue, histological characteristics have been also quantitated. According to that, RFA of all implant systems in both study and control groups in type IV bone showed the superiority of the BLX design. Therefore, the use of this novel system could be preferred in the presence of a poor bone quality at the implant recipient site, regardless of the presence and/or height of the cortical bone.

It is obvious that the implant geometry can increase the surface area of support. A threaded design implant has 30 to 200% greater surface area compared with a cylinder implant of the same size. Therefore, the threaded implant in poorer density bone is strongly encouraged [30, 31]. Considering this, the use of the BLX design in type III and type IV bone could be a feasible option to ensure a mechanical anchorage thanks to its greater surface area. Besides that, the implant neck design of the TE design which applies additional transversal compression forces to the marginal bone could be also favored if the cortical bone is present.

From another clinical point of view, if the removal of an implant is indicated, double thread design and the presence of two sharp and highly engaging threads at its apex could complicate the removal of a partially or fully integrated BLX implant from a type I and type II bone and a trephine or surgical bur technique should be performed, which might not be the most suitable option in terms of maintaining of the bone and minimizing damage to vital structures [32].

## Conclusion

BLX implant systems could be an appropriate option in the lack of cortical bone at the implant recipient site. This fact is more likely of clinical importance for implant insertions at the posterior maxilla and could be preferred in cases where type IV bone exists. Additionally, both TE and BLX systems could ensure sufficient initial stability. However, further studies are needed to exactly clarify the clinical advantages of the BLX design.

## Data Availability

All data is available in the archive of the Research Laboratories, Christian Albrechts University, Department of Oral and Maxillofacial Surgery.

## Abbreviations

BLT: Bone Level Tapered
SP: Standard Plus
TE: Tapered Effect
ISQ: Implant stability quotient
BA: Bone area
TA: Total area
NBF: Neutral buffered formalin
RFA: Resonance frequency analysis
USA: United States of America
